# The roles of the small nucleolar RNA host gene family in ovarian cancer

**DOI:** 10.1080/15384047.2025.2574773

**Published:** 2025-11-07

**Authors:** Tao Zhang, Dapeng Wu, Zhongjie Li, Wei Han, Jie Shi, Anzhen Chen, Wenjing Zhu

**Affiliations:** a Department of Gynecology, Qingdao Hospital, University of Health and Qingdao Hospital and Rehabilitation Sciences (Qingdao Municipal Hospital), Qingdao, People's Republic of China; b Department of Oncology, Qingdao Hospital, University of Health and Rehabilitation Sciences (Qingdao Municipal Hospital), Qingdao, People's Republic of China; c Qingdao Medical College of Qingdao University, Qingdao, Shandong, People's Republic of China; d Respiratory Disease Key Laboratory of Qingdao, Qingdao Hospital, University of Health and Rehabilitation Sciences (Qingdao Municipal Hospital), Qingdao, People's Republic of China; e NMPA Key Laboratory for Quality Research and Evaluation of Traditional Marine Chinese Medicine, Qingdao, People's Republic of China; f National institution of Drug clinical trial of Qingdao Hospital, University of Health and Rehabilitation Sciences (Qingdao Municipal Hospital), Qingdao, People's Republic of China; g Medical Research Department, Qingdao Hospital, University of Health and Rehabilitation Sciences (Qingdao Municipal Hospital), Qingdao, People's Republic of China

**Keywords:** Ovarian cancer; snoRNA, SNHGs, diagnosis, prognosis

## Abstract

Ovarian cancer is one of the most malignant tumors in women. Long noncoding RNAs have been demonstrated to regulate multiple biological processes, including cell proliferation, migration, apoptosis, and drug resistance, in various cancers. Small nucleolar RNA (snoRNA) host genes (SNHGs) are a group of long noncoding RNAs. Studies have reported that SNHGs are aberrantly expressed in many kinds of cancers and are associated with poor patient prognosis. In ovarian cancer, SNHGs play critical roles in the development and progression of ovarian cancer via different pathways. However, there is a lack of systematic reports on the research progress of SNHGs in ovarian cancer. Therefore, we reviewed the studies on the roles of SNHGs in the early diagnosis, development, and treatment of ovarian cancer and explored the underlying mechanisms to provide new insights into the treatment of ovarian cancer.

## Background

Ovarian cancer, including common and rare ovarian cancer,^1^ is the most malignant tumor in the female reproduction system.^2^ The most common and malignant is high grade serous ovarian cancer and endometrioid cancer, whereas rare ovarian cancers originate from many different cell types, such as germ cell, sex cord-stromal, or mixed types.^3^ More than 75% of ovarian patients were diagnosed at advanced stage due to its concealed location and lack of specific biomarkers for early diagnosis.^4^ Although there are great advances in the surgical treatment and adjuvant chemotherapy for ovarian cancer, the overall survival is still relatively short. Chemoresistance is the reason for the poor prognosis and recurrence of ovarian cancer. More than 80% of patients will experience recurrence within two years after the initial treatment is complete.^5^ Therefore, novel diagnostic biomarkers and target molecules for the early diagnosis and treatment of ovarian cancer are urgently needed.

Noncoding RNAs are a class of RNAs without protein-coding functions; however, they have been reported to play vital roles in the transcription, translation, and modification of genes. As transcripts of more than 200 nucleotides that lack protein-coding ability, long noncoding RNAs (lncRNAs) have been demonstrated to modulate cell proliferation, migration, and apoptosis, as well as drug resistance, by regulating the translation, modification, and differentiation of cancer cells.^6-8^ Among them, lncRNA SNHGs, the group of host genes of small nucleolar RNAs (snoRNAs), are long noncoding RNAs. Many studies have reported the aberrant expression of SNHGs in various tumors.^9-18^ For example, some of the SNHGs, including *SNHG1*, *SNHG3*, *SNHG4*, *SNHG5*, *SNHG6*, *SNHG7*, *SNHG8*, were reported to facilitate the proliferation, invasion and migration of hepatocellular carcinoma cells by activating downstream signaling molecules via miRNA and its target genes.^12^

In ovarian cancer, many SNHGs, including *SNHG1*, *SNHG3*, *SNHG6*, *SNHG7*, *SNHG8*, *SNHG12*, *SNHG13*, *SNHG14*, *SNHG15*, *SNHG16*, *SNHG17*, *SNHG20*, and *SNHG22*, are aberrantly highly expressed, while *SNHG2*, *SNHG9,* and *SNHG10* are lowly expressed,^15^ the locations on chromosomes and some transcriptome structures of SNHGs are in Additional Figure 1. SNHGs can mediate the proliferation, cell cycle progression, invasion, migration, and chemoresistance of ovarian cancer cells by interfering with the expression of microRNAs, as well as influencing the EMT and related signaling pathways, providing new insights into the diagnosis and treatment of ovarian cancer.^15^^,^^19^ However, there is still a lack of reviews on the research progress of SNHGs in ovarian cancer until now.

**Figure 1.**
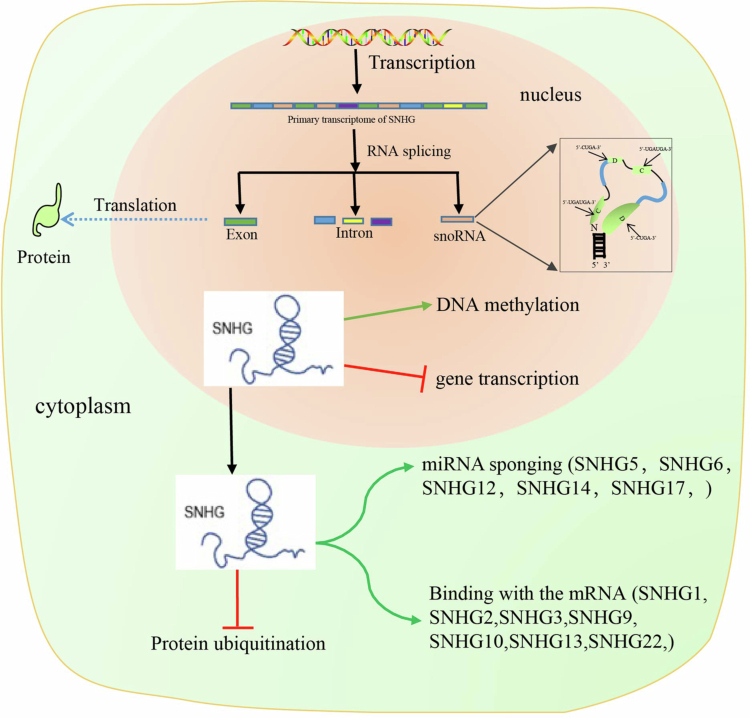
The biogenesis and functions of SNHGs. The hydrolysis products of SNHGs in the nucleus include exons, introns and snoRNAs. SNHGs in the nucleus and cytoplasm have different mechanisms in regulating cancer genesis and progression.

In this article, we reviewed the research progress of SNHGs in the occurrence, development, diagnosis, and treatment of ovarian cancer and revealed the role of SNHGs in ovarian cancer and the underlying mechanisms comprehensively, providing new ideas and methods for the early diagnosis and treatment of ovarian cancer.

## 1. Small nucleolar RNA host genes (SNHGs)

Small nucleolar RNA host genes (SNHGs) are long noncoding RNAs that are widely distributed in the nucleus and cytoplasm of many cells. SNHGs play critical roles in the progression of various cancers, such as breast cancer,^20^ liver cancer,^10^ pituitary adenomas,^21^ gastric cancer,^13^^,^^22^ colon cancer,^23^ lung cancer,^24^ pancreatic adenocarcinoma,^25^ thyroid cancer,^26^ etc. Interestingly, SNHGs in the nucleus and SNHGs in the cytoplasm have different mechanisms in regulating cancer genesis and progression.^15^ The functions of SNHGs in the nucleus involve two pathways, including influencing DNA methylation through the modulation of methylation enzymes and repressing gene transcription through interactions with transcription factors. However, the SNHGs in cytoplasm play different roles via three other pathways: miRNA sponging and the release of miRNA targets, directly combining with mRNA to inhibit its translation and inhibiting the ubiquitylation of single proteins or protein complexes (Figure 1).

The primary transcriptome of SNHGs include exons, introns and snoRNA, which plays an important role in the process of post-transcriptional modification, ribosomal RNA cleavage, RNA silencing and alternative splicing, and telomerase maintenance.^27^ The hydrolysis products of each SNHG include one or more snoRNAs, for example, 8 snoRNAs, including snoRNA22, and snoRNA25−31, were embedded in *SNHG1.*^28-30^ Currently, several studies have reported that SNHGs can affect the occurrence, development, and prognosis of tumors by interacting with the internal snoRNAs.^31^ Among these SNHGs, *SNHG1* play a critical role in many malignant tumors, including colon cancer,^32^^,^^33^ ovarian cancer,^34^^,^^35^ pancreatic cancer,^36^^,^^37^ glioma,^38^^,^^39^ and lung cancer,^40^^,^^41^ via multiple pathways and mechanisms. In ovarian cancer, 17 SNHGs reportedly participate in the proliferation of carcinoma cells, apoptosis, invasion, migration, drug resistance, and prognosis via different regulatory mechanisms.^42-48^

## 2. Effect of SNHGs on ovarian cancer and the underlying mechanisms

**Figure 2.**
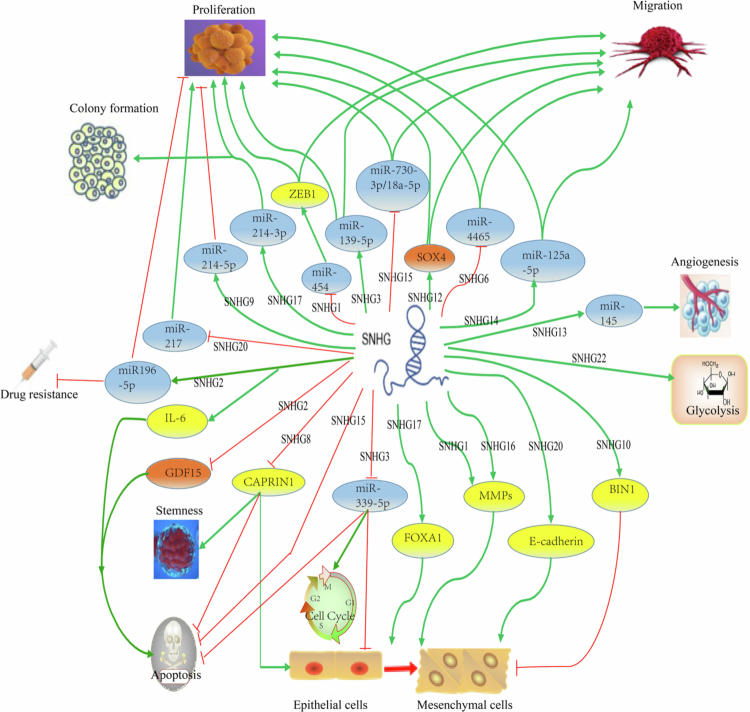
Mechanisms of SNHGs in ovarian cancer. SNHGs can affect the biological behaviors of ovarian cancer cells by regulating the expression of miRNAs or protein molecules, such as genes related to proliferation, cell cycle progression, invasion, migration, and chemoresistance. ZEB1: zinc finger E-box-binding homeobox 1, SOX4: SRY-box transcription factor 4, BIN1: bridging integrator 1, MMPs: matrix metalloproteinases, FOXA1: fork head box A1, CAPRIN1: cell cycle-associated protein 1, GDF15: growth differentiation factor 15, IL−6: interleukin−6 receptor.

SNHGs play a vital role in promoting or inhibiting ovarian cancer via various pathways and multiple network regulatory mechanisms (Table 1). Several kinds of SNHGs, including *SNHG1*, *SNHG3*, *SNHG6*, *SNHG7*, *SNHG8*, *SNHG12*, *SNHG13*, *SNHG14*, *SNHG15*, *SNHG16*, *SNHG17*, *NHG20,* and *SNHG22*, are overexpressed in the early stage of ovarian cancer, and they promote the occurrence and development of ovarian cancer by influencing protein methylation, epithelial‒mesenchymal transition (EMT) and cell cycle arrest as well as promoting angiogenesis (Figure 2).

**Table 1.** The effect of SNHGs on the biological behaviors of ovarian cancer cells.

SNHGs	Chromosomal location	Clinical manifestations	Targeted miRNAs	Cell lines	Effect	References
*SNHG1*	11q12.3	Cell proliferation, invasion, apoptosis and metastasis	miR−454	A2780, OCC1, H8710, SKOV3	Promotion	[35,49,50]
*SNHG2*	1q25.1	Apoptosis, prognosis, proliferation, cisplatin resistant	miR−196-5p	Caov3, OVCAR4, OVCAR5, OVCAR8, EFO27, MCAS, SKOV3, A2780, HO8910, HEY	Inhibition	[51-53]
*SNHG3*	1p35.3	Proliferation, invasion, prognosis, migration, drug resistance, G1/G0 arrest	miR−139-5p, miR−339-5p	SKOV3, OVCAR3, A2780, ES2, OV90, TOV21G, HeyA8	Promotion	[54-57]
*SNHG5*	6q14.3	Paclitaxel sensitivity	miR-23a	SKOV3, MeyA−8	Inhibition	[58,59]
*SNHG6*	8q13.1	Proliferation, migration	miR−4465	HEK293T, ES2, RMG1, TOV21G, OVCA420, OVISE	Promotion	[42,43,60]
*SNHG7*	9q34.3	Paclitaxel resistant	miR−3127-5p	SKOV3/PTX, heyA8/PTX, A2780	Promotion	[44,61]
*SNHG8*	4q26	Proliferation, migration, EMT, stemness	–	SKOV3, ES2, CaOV3	Promotion	[45]
*SNHG9*	16p13.3	Proliferation, migration, invasion	miR−214-5p	SKOV3, OVCAR3, A2780, IOSE‐80	Inhibition	[46]
*SNHG10*	14q32.13	Proliferation, colony formation, migration, and invasion	miR-200a-3p	A2780, SKOV3, OVCAR3, OV90	Inhibition	[47]
*SNHG12*	1p35.3	Proliferation, migration, immune escape	miRNA−129	HEK293T, SKOV3, THP1, A2780, PA1, HO8910	Promotion	[62,63]
*SNHG13*	4q12	Angiogenesis, proliferation, migration, invasion	miR−145	P4936, PC3, DU145, OVCA432, TOV112D, HO8910	Promotion	[64,65]
*SNHG14*	15q11.2	Proliferation, cell cycle	miR-125a-5p	SKOV3, IOSE80	Promotion	[66,67]
*SNHG15*	7p13	Proliferation, G1/G0 arrest, apoptosis, migration	miR−370-3p miR-18a-5p	A2780, CAOV3, SKOV3, OVCA433, ES2, HO8910, OMC685	Promotion	[68,69]
*SNHG16*	17q25.1	Proliferation, migration, invasion, paclitaxel-resistance	–	A2780, OVCAR3, SKOV3, CAOV39	Promotion	[70,71]
*SNHG17*	20q11.23	Prognosis, proliferation invasion	miR−214-3p	OVCAR3, PEO1, SKOV−3, A2780, Caov−3	Promotion	[48,72]
*SNHG20*	17q25.2	Proliferation, migration, invasion, EMT	miR−217	SKOV3, OVCA429, OVCA433, OVCAR3, A2780	Promotion	[73-75]
*SNHG22*	18q21.1	Cisplatin- and paclitaxel-resistance, glycolysis	miR−2467	Hey, OAW28, COV362, OVCAR3, CAOV3, SKOV3, A2780, ES2, HO8910	Promotion	[76,77]

### 2.1 SNHGs promote or inhibit ovarian cancer via microRNA

SNHGs serve as oncogenes by influencing the proliferation and migration ability of ovarian cancer cells. It has been reported that many SNHGs are aberrantly highly expressed in ovarian cancer tissues, which can promote the growth, invasion, and metastasis of ovarian cancer, and silencing these SNHGs can have opposite effects in vitro.^15^^,^^19^^,^^34^^,^^42^ For instance, *SNHG1* can upregulate zinc finger E-box-binding homeobox 1 (ZEB1) by inhibiting the expression of miR−454 and then promoting the proliferation and migration of ovarian cancer,^35^ as shown in Additional Figure 2. Similarly, *SNHG2* has been demonstrated in the ovarian granulosa cell tumor cell line KGN, where it leads to the upregulation of IL6 and increases the apoptosis rate of these cells.^78^ In ovarian clear cell carcinoma, *SNHG6* influences the expression of the enhancer of EZH2 by sponging miR−4465, thus promoting cell proliferation and migration.^43^ Interestingly, *SNHG12* can promote the expression of the transcription factor SOX4 in ovarian cancer tissues and increase the proliferation and migration of tumor cells.^62^ *SNHG14* is overexpressed in ovarian cancer tissues and is negatively associated with the overall survival of patients. *SNHG14* not only promotes the proliferation and migration of ovarian cancer cells by regulating the expression of microRNA-125a-5p but also promotes tumor progression by regulating the expression of DiGeorge syndrome critical region 8 (DGCR8).^66^ *SNHG15* also promotes the proliferation of ovarian cancer cells through upregulated CDK6 via sponging miR−370-3p.^68^ *SNHG20* promotes cell proliferation and invasion by suppressing the expression of miR−217 in ovarian cancer.^75^

Moreover, SNHGs can interfere with the cell cycle of ovarian cancer cells via microRNAs. In ovarian cancer, *SNHG3* promotes the expression of TRPC3 by interfering with miR−339-5p and then influences the cell cycle and subsequently inhibits tumor cell apoptosis. In addition, a study showed that knockdown of the *SNHG3* gene can induce cell cycle arrest in the G0/G1 phase in vitro, thus promoting cell apoptosis.^57^ Interestingly, *SNHG15* knockdown also induced G1/G0 phase arrest and apoptosis.^68^ *SNHG17* facilitates the expression of CDK6 (a cell cycle regulator) via molecular sponges for miR−214-3p and results in the proliferation of carcinoma cells and the formation of new clones.^48^

Furthermore, SNHGs can influence angiogenesis and ATPase activity and serve as proto-oncogenes to promote the progression of ovarian cancer. *SNGH13* is overexpressed in ovarian cancer patients at an advanced stage accompanied by multiple metastases.^79^ *SNHG13* can directly combine with miR−145 to regulate the expression of VEGF and then promote the formation of new blood vessels in tumors.^64^ In addition, *SNHG3* interferes with miR−139-5p and then promotes the expression of Notch1 to promote the occurrence and development of ovarian cancer, thus serving as a proto-oncogene.^55^ Energy supply is critical for tumor metastasis, and *SNHG22* regulated by SP1 can promote glycolysis and the proliferation of cancer cells, thus facilitating the metastasis of ovarian cancer.^77^

In addition, SNHGs can also suppress ovarian cancer via microRNAs. Three SNHGs, including *SNHG2*, *SNHG9,* and *SNHG10*, have been found to play a protective role in ovarian cancer.^46^^,^^47^^,^^51^ *SNHG2* was downregulated in the ovarian tissues versus normal ovarian tissues, and the expression of *SNHG2* is closely related to the clinical staging, pathological types, and prognosis of the ovarian cancer patients.^80^ It has been shown that *SNHG2* can block the transcription of CCAAT/enhancer binding protein *β* (CEBPB)-mediated growth differentiation factor 15 (GDF15), further leading to the apoptosis of ovarian cancer cells.^51^ *SNHG2* can also suppress ovarian cancer cell proliferation by regulating the expression of homeobox A5 (HOXA5) through miR−196-5p.^52^ *SNHG9* suppresses the progression of ovarian cancer cells by regulating miR−214-5p/CRY2 axis, acting as a tumor suppressor gene.^46^ Another study showed that the low expression of *SNHG10* is also associated with poor prognosis of ovarian cancer patients.^47^ Mechanically, *SNHG10* combines with miR-200a-3p to form an RNA-induced silencing complex (RISC), which can act with tumor suppressor bridging integrator−1(BIN1), subsequently suppressing the proliferation and EMT of ovarian cancer cells.^47^

### 2.2 SNHGs influence the progression of ovarian cancer via EMT and EMT-related signaling pathways

SNHGs can affect the invasion and migration of ovarian cancer cells via interfering with EMT, EMT-related signaling pathways as well as the stemness of tumor cells.^8^^,^^19^^,^^34^^,^^45^^,^^47^

Recent study showed that *SNHG1* and *SNHG16* can regulate the activity of matrix metalloproteinases (MMPs) to promote EMT, and then influence the invasion and migration abilities of ovarian cancer cells.^34^^,^^70^ *SNHG10* can upregulate tumor suppressor bridging integrator−1 (BIN1) to inhibit tumor cell proliferation and EMT. The knockdown of *SNHG15* can significantly suppress the migration and invasion of epithelial ovarian cancer cells by increasing the expression of miR-18a-5p,^69^^,^^81^ and *SNHG17* has been reported to promote EMT in ovarian cancer cells by upregulating fork head box A1 (FOXA1).^72^

In addition, SNHGs can affect the EMT properties of ovarian cancer cells via *β*-catenin. *SNHG3* can regulate the development and progression of ovarian cancer cells and affect the diagnosis of ovarian cancer patients via the GSK3/β-catenin pathway.^54^ After silencing *SNHG3*, the expression of some metastasis-related proteins, including CDK1, MMP3, and MMP9, is evidently reduced, suggesting that *SNHG3* influences protein expression in ovarian cancer tissues to affect the migration and metastasis of tumor cells.^54^^,^^56^^,^^57^ *SNHG8* can combine with cell cycle-associated protein 1 (CAPRIN1) to influence the Wnt/β-catenin pathway, promoting the proliferation, migration, and EMT of ovarian cancer cells and suppressing cell apoptosis and stemness.^45^ *SNHG20* has been reported to be upregulated in serous epithelial ovarian cancer and induce the expression of E-cadherin by activating the Wnt/β-catenin pathway, subsequently promoting the proliferation, migration, and EMT ability of ovarian cancer cells.^73^^,^^82^

## 3. Prospects of SNHGs in the treatment of ovarian cancer

**Figure 3.**
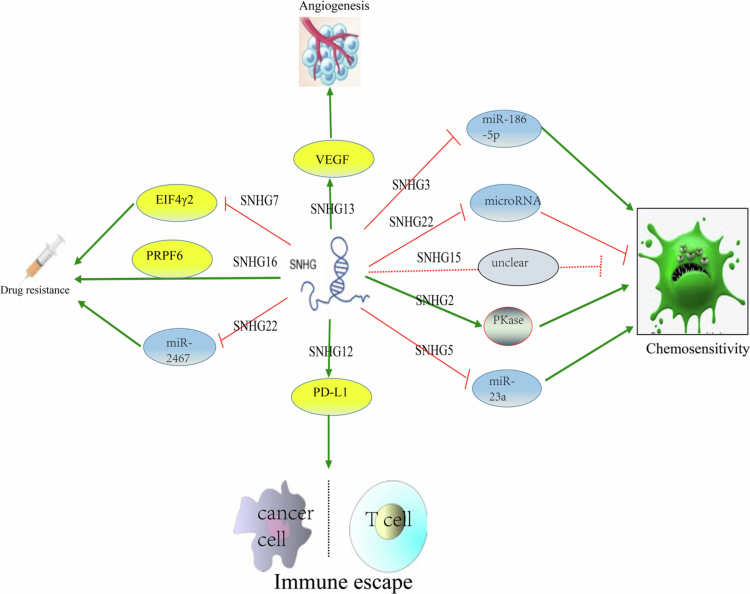
Prospects of SNHGs in the treatment of ovarian cancer. Based on the role of SNHGs in ovarian carcinogenesis and progression, SNHGs could play an important role in ovarian cancer treatment via regulating the expression of miRNA or protein molecules. VEGF: vascular endothelial growth factor, PD-L1: programmed death-ligand 1, PRPF6: Pre-mRNA processing factor 6, EIF4γ2: eukaryotic translation initiation factor 4 *γ*-2.

The important role of SNHGs in various biological processes related to carcinogenesis, together with their cancer-specific expression patterns, has made SNHGs promising therapeutic targets. Therefore, many strategies have been explored for their roles in the treatment of ovarian cancer.^83^ Generally, there are two main approaches, which have already been applied for targeting different SNHGs in OC: to alter their expression level or to inhibit their interactions with other macromolecules.^84^ In the treatment for ovarian cancer, satisfied tumor cell debulking surgery and effective chemotherapeutic drugs played important roles in progression-free survival and overall survival.^5^^,^^85-90^ According to clinical guidelines and expert consensus,^86^ the common chemotherapeutic drugs for ovarian cancer include cisplatin, paclitaxel, tamoxifen, doxorubicin, fluorouracil, etc. It has been reported that SNHGs can influence the sensitivity of ovarian cancer cells to chemotherapeutic drugs, mediate immune escape, and regulate tumor angiogenesis to influence the therapeutic effects on cancer (Figure 3).

### 3.1 SNHGs can influence the chemosensitivity of ovarian cancer cells to many drugs through multiple pathways

Several SNHGs have been documented to influence the chemosensitivity of ovarian cancer cells to cisplatin.^53^^,^^76^^,^^81^ *SNHG2* can enhance the chemosensitivity of ovarian cancer cells to cisplatin via activating protein kinase pathways, while *SNHG22* can suppress the chemosensitivity of ovarian cancer cells to cisplatin via interacting with microRNAs. *SNHG15* has a similar function to that of *SNHG22*, but its underlying mechanism is still unclear.^15^^,^^81^^,^^91^^,^^92^

SNHGs can affect the sensitivity of ovarian cancer cells to paclitaxel. It has been reported that *SNHG5* enhances the sensitivity of ovarian cancer cells to paclitaxel via sponging miR-23a.^58^ *SNHG7* mediates paclitaxel resistance in ovarian cancer cells by interacting with eukaryotic translation initiation factor 4 *γ*-2 (EIF4γ2), while after silencing the expression of *SNHG7*, it can restore paclitaxel sensitivity in tumor cells.^44^ Pre-mRNA processing factor 6 (PRPF6) promotes the paclitaxel resistance of ovarian cancer by regulating *SNHG16.*^71^ *SNH22* influences the expression of galectin−1 (Gal−1) via interacting with miR−2467, mediating the drug resistance of cisplatin/paclitaxel during chemotherapy.^76^

In addition, SNHGs can influence the endocrine therapy of ovarian cancer cells. Li et al. found that *SNHG3* mediates the sensitivity of ovarian cancer cells to tamoxifen by influencing glycolysis, the Krebs cycle and oxidative phosphorylation and inhibiting the expression of miR‐186‐5p.^56^

Moreover, SNHGs can influence the immune escape and angiogenesis of ovarian cancer cells during chemotherapy. Mechanically, *SNHG12* facilitates IL-6R transcription/expression via the recruitment of NF-κB and induces the expression of programmed death-ligand 1 (PD-L1) on the surface of M2 macrophages; subsequently, the proliferation of T cells is suppressed, leading to the immune escape of ovarian cancer cells.^63^ Research has shown that *SNHG13* can act as a therapeutic target in ovarian cancer because *SNHG13* can regulate the expression of vascular endothelial growth factor (VEGF) via miR−145 and promote tumor angiogenesis.^64^

## 4. SNHGs and the prognosis of ovarian cancer

Ovarian cancer has the highest mortality rate among all gynecologic malignancies, and there are no specific and effective biomarkers to predict its prognosis. To date, many SNHGs have been reported to be related to the prognosis of ovarian cancer. Among them, *SNHG3*, *SNHG6*, *SNHG15*, *SNHG16,* and *SNHG20* are aberrantly upregulated in ovarian cancer, and their expression levels are positively associated with multiple clinical factors, including tumor size, depth of invasion, clinical analysis, remote metastasis, pathological types, tissue differentiation of ovarian cancer, suggesting that the above SNHGs are associated with the poor prognosis of ovarian cancer.^43^^,^^54^^,^^81^^,^^82^^,^^93^^,^^94^ However, patients with low *SNHG2* expression had shorter overall survival and progression free survival, and the expression level of *SNHG2* was negatively associated with tumor size, depth of invasion and clinical staging, suggesting that *SNHG2* could be an indicator of good prognosis of ovarian cancer.^80^ According to reported studies, in addition to *SNHG2*, *SNHG5*, *SNHG9,* and *SNHG10*, most SNHGs play a role as a proto-oncogene in the occurrence and progression of ovarian cancer via different pathways.^42^^,^^46^^,^^64^^,^^80^ Based on the current research, SNHGs take part in the progression of ovarian cancer in various pathways, and it can combine several factors, like CA125 and tumor size, to construct a precise predictive model for ovarian cancer.^70^^,^^95-98^

## 5. Conclusion

Above all, the dysregulation of SNHGs were significantly associated with the stage, migration, invasion, and poor diagnosis of ovarian cancer. Many studies have shown that SNHGs can regulate the progression of various tumors via activating different signaling pathways, interacting with snoRNAs and acting as sponge miRNAs. SNHGs can act as independent prognostic factors for ovarian cancer. However, the regulatory networks of SNHGs are complex, and their underlying mechanisms are still unclear. Therefore, further exploration of the mechanisms of SNHGs and snoRNAs is necessary to provide new insights and novel targets for ovarian cancer.

## Consent for publication

Not applicable.

## Abbreviations

ATPase: Adenosine triphosphatase
BIN1: Bridging integrator 1
CA125: Cancer antigen 125
CEBPB: CCAAT/enhancer binding protein β
CAPRIN1: Cell cycle-associated protein 1
CRY2: Cryptochrome circadian regulator 2
CDK: Cyclin-dependent kinases
DGCR8: DiGeorge syndrome critical region 8
EMT: Epithelial mesenchymal transformation
EIF4γ2: Eukaryotic translation initiation factor 4 *γ*-2
FOXA1: Fork head box A1
Gal−1: Galectin−1
GSK3: Glycogen synthase kinase 3
GDF15: Growth differentiation factor 15
HOXA5: Homeobox A5
IGF2: Insulin-like growth factor 2
IL-6R: Interlukin−6 receptor
lncRNA: Long non-coding RNA
MMPs: Matrix metalloproteinases
NF-κB: Nuclear factor kappa-B
snoRNA: Nucleolar small molecule RNA
PRPF6: Pre-mRNA processing factor 6
PD-L1: Programmed death-ligand 1
rRNA: Ribosomal RNA
RISC: RNA-induced silencing complex
snoRNPs: Small nucleolar ribonucleoproteins particles
SNHG: Small nucleolar RNA host genes
SOX4: SRY-Box transcription factor 4
TRPC3: Transient receptor potential 3
VEGF: Vascular endothelial growth factor
EZH2: Zester homolog 2
ZEB1: Zinc finger E-box-binding homeobox 1
